# The influence of culture on care receivers’ satisfaction and aggressive tendencies in the emergency department

**DOI:** 10.1371/journal.pone.0256513

**Published:** 2021-09-02

**Authors:** Alon Lisak, Dorit Efrat-Treister, Ella Glikson, Vladimir Zeldetz, Dan Schwarzfuchs

**Affiliations:** 1 Department of Management, Ben-Gurion University of the Negev, Be’er Sheva, Israel; 2 The Graduate School of Business Administration, Bar-Ilan University, Ramat Gan, Israel; 3 Department of Emergency Medicine, Soroka University Medical Center, Be’er Sheva, Israel; Georgia Southern University, UNITED STATES

## Abstract

**Introduction:**

Reducing aggressive tendencies among care receivers in the emergency department has great economic and psychological benefits for care receivers, staff, and health care organizations. In a study conducted in a large multicultural hospital emergency department, we examined how cultural factors relating to ethnicity interact to enhance care receivers’ satisfaction and reduce their aggressive tendencies. Specifically, we explored how care receivers’ cultural affiliation, individual cultural characteristics, and the cultural situational setting interact to increase care receivers’ satisfaction and reduce their aggressive tendencies.

**Method:**

Data were collected using survey responses from 214 care receivers. We use structural equation models and the bootstrap method to analyze the data.

**Results:**

Care receivers’ openness to diversity (an individual cultural characteristic) was positively related to their satisfaction that was associated with lower aggressive tendencies, only when they were affiliated with a cultural minority group and when the cultural situational setting included language accessibility.

**Conclusion:**

Our results demonstrate that cultural affiliation, individual cultural characteristics, and cultural situational setting can affect care receivers’ satisfaction and aggressive tendencies in a multicultural emergency department context. In particular, high cultural openness of care receivers, and making information accessible in their native language, increased satisfaction and reduced aggressive tendencies among cultural minority care receivers in our study.

## Introduction

In any organization, aggression against employees can elevate levels of turnover, exhaustion, and burnout [1–4], can increase the frequency of errors, and may impair staff performance [5–7]. Extreme forms of aggression even cause physical injuries and deaths [8]. This is equally true for aggression against medical staff in health care systems, making this a severe problem with substantial costs and implications [9].

Although care receivers (patients and their escorts) have been found to engage in aggression against medical staff in all hospital areas, some settings are at higher risk. Consistent findings indicate that medical staff in emergency departments (EDs) are at extreme risk of being the target of aggression, compared to medical staff in other hospital wards [10, 11]. Landau and Bendalak found that 75% of ED staff members reported experiencing aggression from care receivers daily over the past six months [12]. Such findings reflect the unique characteristics of the ED, the “entryway” to the hospital, which is characterized by high levels of crowding and long wait durations that can heighten care receivers’ aggressive tendencies [13–15].

Despite the severe implications of aggression targeted at care providers in the ED, antecedents (and buffers) of such aggression are under-studied and poorly understood [16–18]. In the present study, we focus on aggressive tendencies, based on existing findings showing that aggressive tendencies often predict actual aggressive behavior [19–21]. These tendencies are defined as a propensity to engage in low-level aggressive behavior, such as yelling, cursing, verbally abusing staff, damaging equipment, or interfering with work processes [14]. Understanding the factors that trigger and (equally important) inhibit aggressive tendencies among care receivers, is crucial for developing ways of curtailing such tendencies before they escalate into more severe aggression.

Customer satisfaction, defined as the extent to which the service customers receive is congruent with their expectations, is associated with lower aggressive tendencies [22–25]. In the health care context, care receivers’ satisfaction has an additional critical attribute, in that the service provided may have a major impact on the physical well-being of the patient or a loved one. Hence, a bad service experience in the health care context constitutes a potential threat, increasing the care receiver’s anxiety and stress—conditions that can set the stage for aggression. Studies in the medical context suggest that when care receivers are dissatisfied, aggression can become their mode of communication with medical staff, especially in situations that are already characterized by communication difficulties, such as language barriers [26–28]. Equally, higher satisfaction among care receivers is associated with reduced aggressive tendencies in such contexts [29].

Given the relationship between care receivers’ satisfaction and aggressive tendencies, an essential question is how to enhance satisfaction in health care contexts, and especially in the ED, where sources of stress add to the anxiety naturally experienced by patients and their escorts, and where opportunities for miscommunication are manifold. Our study consider culture as a potential influence on care receivers’ satisfaction and subsequently on their aggressive tendencies.

In general terms, culture is defined as a collective programming of the mind that distinguishes the members of one group from others [30]. Individuals act within a cultural system that defines certain clusters of behaviors which go together, including beliefs, communication norms, and language [31]. One key source of cultural difference in many societies is ethnicity and the culture of ethnic minority groups can differ significantly from the culture of the ethnic majority group [31]. Further, this is a source of cultural difference that has grown in importance over recent years, as many countries have become more ethnically diverse—a product of growing globalization, political changes, migration, or changes in birth patterns of different demographic groups [32, 33].

This growing diversity means that the (already stressful) interaction between care receivers and ED medical staff often involves cross-cultural communication, where staff members and care receivers may not only belong to different ethnic cultural groups, but may also have different native languages. Indeed, the communication difficulties that naturally arise from ED realities (such as the multitasking and time pressure experienced by medical staff) become even more complex when medical staff and care receivers differ in their culture, and especially in their native language. It thus behooves us to ask whether and how culture-embedded factors, such as cultural characteristics, cultural affiliation, and language accessibility, may influence satisfaction levels and aggressive tendencies of care receivers in such multicultural, multi-ethnic ED contexts.

### Research framework

Our study relies on the Culture-Person-Situation approach (CuPS [31]). Under CuPS, individual attitudes and behaviors reflect an interaction between (a) the individual’s cultural affiliation (Culture); (b) individual cultural characteristics (Person); and (c) the cultural situational setting (Situation). According to the CuPS approach, different cultural logics weave together various scripts, behaviors, practices, and cultural patterns around a central theme, creating logical consistency and serving as a source of meaning for individuals who affiliate with a specific culture, including cultures associated with ethnic groups. Individuals may affiliate with different cultural groups on different levels, including groups based on immutable demographic characteristics (e.g., ethnicity or age) and groups that are chosen (e.g., occupational or organizational cultures). In this paper we focus on affiliation with an ethnic minority group with shared values, beliefs, behavioral norms, and language that differ from those of the ethnic majority group.

Cultural affiliation helps to define psychological situations and create meaningful clusters of behavior according to particular logics [31]. That is, different cultures (e.g., culturally distinguished ethnic groups) emphasize different values, and differ in their underlying assumptions about what is normative, or rather, what violates the norm [34–37]. Therefore, individuals who affiliate with different cultures, can differ in their satisfaction with a given social situation [31]. In addition, cultural affiliation may have implications for individual behaviors in a multicultural context. In the ED, staff generally behave according to the norms of the majority cultural group and use the majority language for communication. Thus, care receivers from minority cultural groups may experience communication difficulties, and as a result, may feel less satisfied with the treatment they receive [38]. There is also evidence that care receivers from minority cultural groups tend to receive fewer explanations and follow-ups from medical staff, compared to care receivers from majority cultural groups [27, 39–42]. These circumstances may lead cultural minority care receivers to feel they are treated with less respect, which could result in lower satisfaction compared to members of majority cultural groups [43, 44].

Individual cultural characteristics include aspects of a person’s motivation, skills, and knowledge that contribute to proficiency in a culturally diverse context [45]. An individual characteristic that is highly relevant in this context is openness to diversity. Openness to diversity (OTD) is defined as the extent to which someone holds a non-judgmental attitude about diverse cultural behaviors and expectations, and a positive perspective regarding cultural differences [46, 47].

Cultural situational setting refers to the presence or absence of cultural cues within the given context that can influence people’s satisfaction. One key aspect of the cultural situational setting is language accessibility—the degree to which a product, service or environment is available to speakers of different languages in a given area, so that they may consume such services in their native language [48]. In the ED, language accessibility—e.g., through interpreter services, signs, and written information [49, 50]—enables care receivers to understand essential medical and health care system information. Hence, ensuring language accessibility in the ED can elevate the satisfaction of diverse cultural care receivers [49].

In a multicultural context, the outcome of any interaction is complicated by cultural disparities between the parties to the encounter. Recent decades have seen growing attention in health care systems to the potential implications of such cultural disparities between care receivers and care providers. In particular, many health care institutions now maintain research into medical cultural competency policies and practices—a set of behaviors, attitudes, and procedures designed to ensure that members of a system or institution can work effectively in cross-cultural situations [51, 52]. Thus far, research into cultural competency practices tends to focus on two main areas: cultural training designed to improve the cultural proficiency of medical staff (e.g., [41, 53–55] and the effects of improved language accessibility (e.g., [56, 57].

While research into cultural competency in health care institutions has provided essential insights, the literature has largely neglected one important factor: the cultural characteristics of the individuals involved, and particularly of care receivers. Like people everywhere, care receivers differ in their individual cultural characteristics, including their ability to communicate with people from other cultures and to get by in a majority culture different from their own. It stands to reason that these characteristics may affect care receivers’ satisfaction and aggressive tendencies in a multicultural ED.

Additionally, most existing studies relate to only one cultural factor (e.g., cultural proficiency, cultural accessibility). Almost no studies have explored the mutual contribution of such cultural factors together, or the interaction between them.

Our work helps bridge these gaps in the literature. Following the CuPS approach [31], we examine how a cultural characteristic of care receivers (specifically, OTD) interacts with their cultural affiliation (member of a minority or majority cultural group) and with situational factors (the presence or absence of language accessibility) to affect their satisfaction and aggressive tendencies in a multicultural ED context.

### Hypothesis development

#### Care receivers’ openness to diversity and satisfaction

Individuals with high OTD tend to view cultural differences as positive. They are curious about other cultures, open to learning from dissimilar others, and culturally tolerant, seeking to understand diverse perspectives and to reduce problems arising from cultural misunderstandings [46, 47, 58–61]. High OTD is associated with low frustration, high trust, good adjustment, and high satisfaction with multicultural interactions [45, 62, 63]. Hence, we expect a positive relationship between care receivers’ OTD and their satisfaction with their experience in a multicultural ED.

#### The moderating role of cultural setting (language accessibility) and cultural affiliation

We suggest that language accessibility and cultural affiliation can facilitate the relationship between care receivers’ OTD and their satisfaction with their experience in the ED. *Language accessibility* in the ED refers to the availability of medical and administrative information in the care receivers’ native language [42, 49, 64]. Language accessibility can reduce language barriers between medical staff and care receivers and is associated with reduction in medical errors, unanswered questions, health inequity and poorer health outcomes [42, 44, 56]. Moreover, language accessibility can facilitate creation of a psychologically safe communication climate, defined as an atmosphere characterized by open, supportive communication based on trust, where all parties feel encouraged to speak up [65, 66]. Care receivers who are high in OTD are better-positioned (compared to those lower in OTD) to take advantage of language accessibility, increasing their satisfaction.

Although information delivery can benefit all care receivers, in the ED—an environment controlled by the cultural majority language and norms [50, 67]—individuals from minority cultural groups are those for whom openness to diversity and language accessibility are most relevant. Taken together, we therefore predict that the relationship between care receivers’ OTD and their satisfaction with the ED will be facilitated by language accessibility (i.e., whether care receivers have access to important information in their native language); and that cultural affiliation will moderate this effect (i.e., the effect will be more significant for care receivers who belong to minority cultural groups).

H1: Care receivers’ openness to diversity will be positively related to their satisfaction with their experience in a multicultural ED context. This relationship will be moderated by language accessibility and cultural affiliation, such that the relationship will be stronger when language accessibility is high and for care receivers from a minority cultural group.

#### Care receiver satisfaction and aggressive tendencies

As described in the introduction to this manuscript, care receivers in hospitals may feel highly vulnerable, uncertain, and under strain, all of which elevate people’s tendency to engage with others in an aggressive manner [12]. Individuals who are less satisfied tend to be more aggressive in various work contexts (see the meta-analysis by Hershcovis et al. [23]), including medical settings [26, 29]. However, as noted earlier, most previous studies examined the satisfaction–aggression relationship indirectly through staff perceptions, rather than measuring care receivers’ tendency to aggress. We aim to test this relation by exploring how care receivers’ satisfaction is related to their tendency to aggress.

H2: Care receivers’ satisfaction will be negatively related to their tendency to aggress against hospital staff in a multicultural ED context.

Previous studies suggest that care receivers from minority groups and those with low proficiency in the majority language express less satisfaction with medical encounters than other care receivers [42]. Cultural competence can enhance their satisfaction by enabling better cross-cultural communication [41]. Following the logic of H1 and H2, we thus expect an indirect negative relationship between care receivers’ OTD and aggressive tendencies, mediated by satisfaction. We also expect that this relation will be stronger when language accessibility is high, and for care receivers from a minority cultural group.

H3: Care receivers’ openness to diversity will be negatively and indirectly related to their aggressive tendencies through satisfaction with their experience in the ED. This relationship will be stronger when language accessibility is high and for care receivers from a minority cultural group.

## Method

### Ethics approval

The study was approved by the Soroka Medical Center Helsinki committee, approval number 0126-16-SOR. (Please see translation of this approval in the file “S2 File.”). Written consent was obtained. No minors were included in this study.

### Participants

The research model in this study explored a three-way interaction between care receivers’ openness to diversity, language accessibility, and cultural group affiliation on satisfaction, and in turn on aggressive tendencies. To find the sample size needed to detect this model, we used G*Power software V.3.1.9 based on a linear multiple regression with a fixed-effects model and regression coefficient with a power of 80%, significance (α) of 5% and at least a medium effect size (Cohen’s *ƒ*^2^ = .06). The results indicate a sample size of 128 care receivers. We added to the sample size an additional 20% to compensate for possible non-responses, producing a minimum of 160 participants. This compensation rate is conservative, as previous studies conducted in similar ED contexts [13, 14] suggest a non-response rate of less than 10%.

We tested our hypotheses among patients and escorts waiting to receive care in an ED of a large public tertiary hospital (level 1 trauma; 1130 beds) located in southern Israel. The hospital’s ED is one of the largest in the country, with the main ED unit receiving more than 150,000 visits annually (more than 400 per day). This ED serves only adult patients and escorts (aged 17 and up).

In Israel, standard policy in any ED is that each patient may be escorted by one individual, who is normally a close relative. Because close relatives tend to be highly invested in the patient’s health, we follow previous studies that explored aggression in Israeli EDs in considering both patients and their escorts as one group of care receivers (see Appendix A in S4 File for a justification of this procedure).

Israel is a multi-ethnic society, with a Jewish majority and a non-Jewish (mainly Arab Muslim) minority, which differ in culture and native language (Hebrew or Arabic). The population of the southern district is culturally diverse, with Jews—the majority cultural group—making up about 73% of the population, Muslim Arabs (nearly all Bedouin) making up about 21%, and members of other groups comprising around 6% [68]. Consistent research indicates that Arabs, and specifically, Bedouin Arabs, consume health services (including ED services) on a larger scale than their relative size in the population [69]. This disproportion is explained by several factors, including high rates of marriage between relatives, high rates of smoking, and a high proportion of diabetics compared to the general population [70–74].

Three groups of care receivers were excluded from the sample: Jewish immigrants with poor Hebrew proficiency, patients who arrived at the ED while drunk or under the influence of drugs, and patients who suffered from dementia. The last two groups received immediate treatment upon registering at the ED, and so were not available in the waiting area to respond to our survey. It is important to note that the proportion of patients presenting with drug or alcohol problems during the time of our study was extremely small (0.001% of all visits).

Finally, we approached only escorts who were with the patient in the waiting room from the start of the patient’s visit, to ensure that all participants in our sample faced the full ED experience.

In short, in this study, we compare the general Jewish majority cultural group (who are using Hebrew as their main communication language) and the Arab minority cultural group (who are using Arabic as their main communication language). The final sample comprised 214 care receivers (85 patients and 129 escorts; Mage = 37.74 years, SDage = 12.01; range 17–69 years; 52% female). Of the total, 40.7% belonged to the Arab minority group and the rest to the Jewish majority group. Ninety-seven percent of the care receivers we approached agreed to respond to the survey.

### Procedure

Data were collected between 8am and 10pm by research assistants. The research assistants worked in teams that generally included at least one Jewish and one Arab data collector, and their interactions with participants were matched by culture. The research assistants approached care receivers at the ED reception desk and gave them an information sheet explaining typical procedures in the ED. The conversation and the written information were in the care receiver’s native language (Hebrew or Arabic). They asked them to read it (when they stood near them) and verify that the information was clear. The data randomization took place such that the condition in which participants were included depended on the exact time of arrival at the ED reception. During every thirty minutes, the condition reversed from with language accessibility to without language accessibility. Between each condition, we stopped data collection for ten minutes, allowing the ED reception room to replace its patients. It is reasonable to assume that the care receivers’ exact arrival time did not depend on any external attribute.

This procedure created two groups: a treatment group of care receivers who were given language-accessible information, and a control group that did not receive this information. The information that we gave to the treatment group was the only information provided to care receivers regarding typical procedures when they arrived at the ED. All care receivers were given specific medical information by their assigned doctors some time after they filled in our surveys.

The data collection proper took place about 90 minutes after care receivers’ arrival, after the patients had met with a nurse and then a doctor for initial triage. The research assistants approached the care receivers waiting in the ED and asked them to respond to a short survey (5–10 minutes) in their native language (only the patient or the escort in each dyad filled in the survey). This survey assessed OTD, satisfaction, aggressive tendencies, cultural affiliation, language accessibility (i.e., whether they had received the information sheet), control variables, and demographics. Respondents indicated the time they had registered at the ED (copied from the file they were given at registration) and the time at which they responded to the survey. This allowed us to match the survey data with hospital records to obtain some of our control variables, such as crowdedness of the ED or time of day. Responses to the survey were anonymous. Participants who completed the survey were thanked and offered a snack as a token of appreciation.

### Measures

All scales were translated and back-translated from English to Hebrew and Arabic (following [75]). All scales were measured on a 7-point Likert-type scale (1 = “very little,” 7 = “very much”).

#### Openness to diversity (OTD)

Was measured using five items based on the OTD scale of Hobman et al. [76] (e.g., “I often spend time with people from cultural groups other than my own”). Cronbach’s alpha was .85.

#### Satisfaction

Was assessed using four items based on the satisfaction scale of Glynn Mangold and Babakus [77] (e.g., “I perceive medical staff as willing to help me”). Cronbach’s alpha was .93.

#### Aggressive tendencies

Were measured through eight items based on the aggressive tendencies measure developed by Efrat-Treister et al. [14]. Sample items: "What are the chances that someone in the ED will hit/…yell at/…curse a care giver.” Cronbach’s alpha was .96.

#### Cultural affiliation

Was obtained through self-reports (Jewish or Arab cultural groups).

#### Language accessibility

Participants indicated whether or not they had received written information in their native language.

#### Controls

Following Carlson and Wu [78], we controlled for age; time already waited in the ED (log-transformed); role (patient or escort); gender; ED shift; and crowdedness of the ED. These variables have all been shown to potentially affect variables linked to aggression in the ED [13, 14].

### Analytical strategy

To test our research model, we employed latent moderated structural equation modeling (LMS) using Mplus 8.2 [79]. As noted by Selig and Preacher [80, p. 147], “using latent variables has the advantage of addressing the problem of measurement error, thus de-attenuating relationships among the constructs.” The first step in our analysis was to test the measurement model using confirmatory factor analysis to verify that the indicators reflected their intended latent variables. We compared a three-factor model of our latent variables (OTD, satisfaction, and aggressive tendencies) with all possible two-factor models and with the one-factor model (in which all items from all three latent variables were collapsed into a single factor). The fit of each model was assessed using two relative fit indices, namely the comparative fit index (CFI; [81]) and the Tucker-Lewis index (TLI; [82]), and two absolute fit indices, namely the chi-squared test and the standardized root mean square residual (SRMR; [83]). We evaluated these fit indexes using the traditional cutoff value of .90 for the CFI and TLI and less than .08 for the SRMR.

The second step of the LMS analysis was to test the relationships between the variables in the structural model. We used a two-step method to assess the overall fit of each LMS model, using maximum likelihood estimation, as recommended by [84]. Under this approach, the log-likelihood ratio test of a null model that does not include the latent interactions is compared with the model that includes the latent interactions to determine whether the parsimonious null model represents a significant loss in fit relative to the more complex latent interaction model. This procedure produces unbiased parameter estimates and is more efficient than other methods [85, 86], such as weighted least squares, which are based on the augmented moment matrix [87]. In our study, the latent interactions are the two-way interactions between OTD and each of the other two cultural variables (language accessibility/cultural affiliation) and the three-way interaction between them.

To test our hypotheses, we proceeded as follows. Hypothesis 1 predicted that care receivers’ OTD would be positively related to satisfaction, and that this relationship would be moderated by language accessibility and cultural affiliation. We tested this hypothesis by examining the three-way interaction coefficient (between OTD, language accessibility, and cultural affiliation) in the presence of the two-way interactions and all other variables. We then conducted a simple slope analysis [88] on the three-way latent interaction, exploring the nature of the interaction between care receivers’ OTD and language accessibility under the various conditions of cultural affiliation. Hypothesis 2, predicting a negative relationship between care receivers’ satisfaction and aggressive tendencies, was tested by examining the coefficients of the relationship between the two variables. Hypothesis 3 predicted a negative and indirect relationship between care receivers’ OTD and aggressive tendencies through satisfaction, with language accessibility and cultural affiliation as moderators. To test this hypothesis, we conducted conditional indirect effect analysis with four conditions (with/without language accessibility X Arab/Jewish cultural affiliation) using the Mplus 8.2. bootstrapping method at 95% bias-corrected confidence intervals with 5000 replications.

## Results

Table 1 presents the means and standard deviations of the study variables, as well as the correlation matrix. To test our predictions, we first fitted the baseline measurement model (model 1), with the three latent constructs (OTD, satisfaction, and aggressive tendencies). This measurement model accurately reproduced the observed covariance matrix (χ^2^_(116_) = 298.09, p < .01; CFI = .94; TLI = .93; SRMR = .058) (see Table 2). All standardized factor loadings of the latent variables on their indicators were significant (p < .01), ranging from .41 to .96. Analyses of the other possible two-factor and one-factor models show a substantial loss of fit relative to the three-factor model (e.g., CFI and TLI < .90 and SRMR > .08 in all these models). A comparison between the models’ chi-squared scores revealed that the three-factor model provides a better fit than all other models (p < .01).

**Table 1:** Means, standard deviations, and intercorrelations of model variables.

Variable	M	SD	1	2	3	4	5	6	7	8	9	10	11	12
1. Age 1	.46	.50	^___^											
2. Age 2	.40	.49	-.76**	^___^										
3. Age 3	.13	.34	-.37**	-.32**	^___^									
4. Time waited	4.60	.78	.02	-.04	.03	^___^								
5. Role	.60	.49	-.07	.02	.07	-.10	^___^							
6. Gender	.50	.50	-.08	.02	.10	-.11	.09	^___^						
7. Shift	.20	.40	-.01	.03	-.02	.01	.06	.09	^___^					
8. Crowding	195.18	23.97	-.02	-.03	.07	.13*	-.01	-.10	-.03	^___^				
9. Cultural affiliation	.41	.49	-.17*	-.04	-.19**	-.12	.11	-.01	.24**	-.12	^___^			
10. Language accessibility	.53	.50	.09	-.12	.05	-.21**	.04	-.05	-.17*	-.01	-.17	^___^		
11. Openness to diversity	4.87	1.19	-.10	-.01	.15*	-.03	.04	.03	.02	.07	-.02	.02	^___^	
12. Satisfaction	5.55	1.32	.03	-.03	-.01	-.13*	.01	.01	-.04	.02	-.04	.12	.21**	^___^
13. Aggressive tendencies	2.77	1.64	-.02	-.05	.07	.18*	-.10	-.09	-.08	.12	-.42**	.01	-.07	-.18**

N = 214

* p < .05

** p < .01.

Notes. Age 1 = 18–29, Age 2 = 30–49, Age 3 = 50–69; Time waited: log was used; Role: Patient = 0, Escort = 1; Gender: Male = 0, Female = 1; Shift: Morning = 0; Afternoon = 1; Cultural affiliation: Jewish = 0, Arab = 1; Language accessibility: No treatment = 0, With treatment = 1.

**Table 2:** Fit indices for measurement model analyses.

Factor and Model	χ^2^	*df*	CFI	TLI	SRMR
Equal form models					
Model 1: Three factors	298.09**	116	.94	.93	.058
Model 2: Two factors (SAT+AGG)	954.35**	118	.71	.66	.159
Model 3: Two factors (SAT+OTD)	626.38**	118	.82	.80	.121
Model 4: Two factors (OTD+AGG)	666.20**	118	.81	.78	.141
Model 5: One factor	1305.05**	119	.58	.64	.211

N = 214; * p < .05

** p < .01.

Notes. OTD = Openness to diversity; SAT = Satisfaction; AGG = Aggression.

The comparisons between Model 1 and Model 2 (Δχ2(2) = 656.26, p< .01), between Model 1 and Model 3 (Δχ2(2) = 328.29, p < .01), between Model 1 and Model 4 (Δχ2(2) = 368.11, p < .01), and between Model 1 and Model 5 (Δχ2(3) = 1006.96, p < .01) were all significant, suggesting better fit for Model 1.

**Fig 1:**
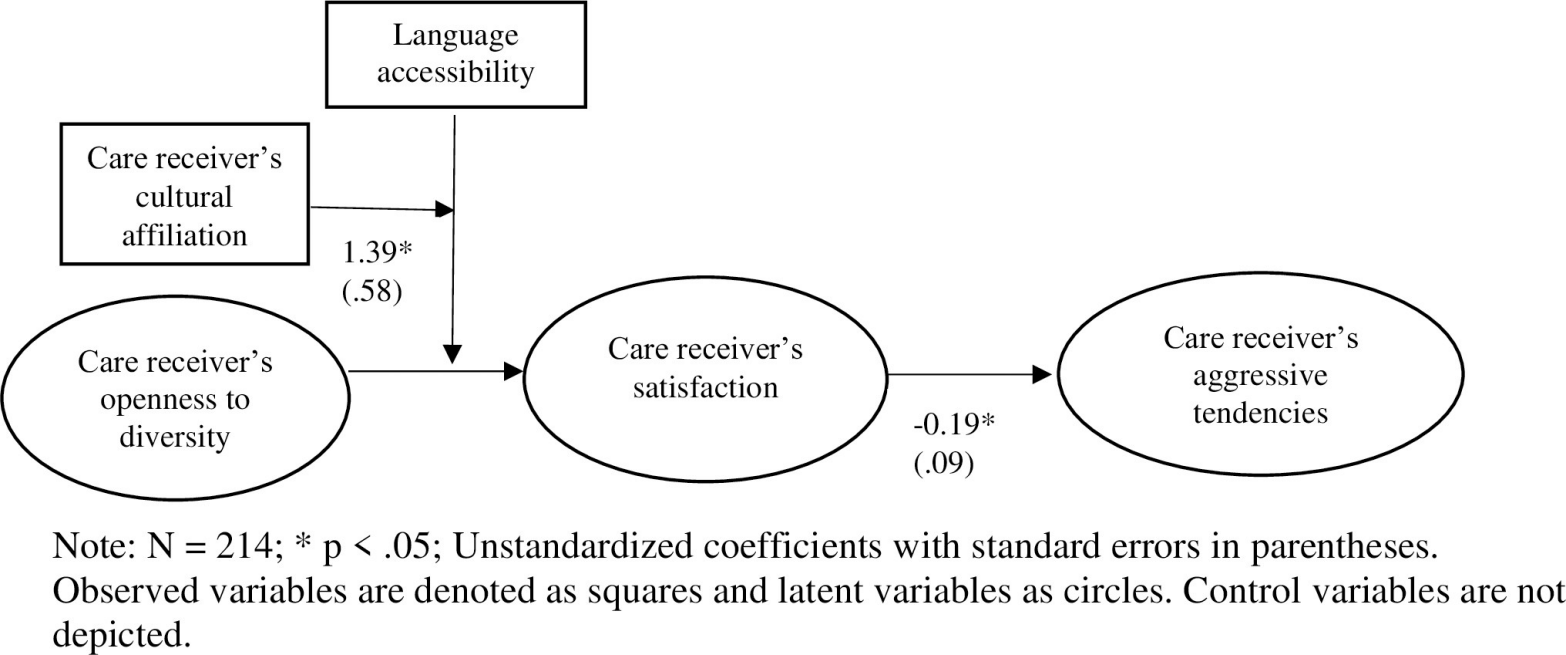
Structural equation analysis for the research model.

Next, we added the latent interactions to the null model (OTD X language accessibility; OTD X cultural affiliation; and the three-way interaction). This model fit the data significantly better than the model without the latent interaction terms (-2 log-likelihood = 5456.40); (Δ-2 log-likelihood = 7.86; χ^2^_(3)_ = 7.86, p < .05). See Table 3 and Fig 1.

**Table 3:** Structural equation analysis for the research model.

	Model 1 3-way interaction on Satisfaction		Model 2 on Aggressive tendencies	
Variable	Estimate	SE	Estimate	SE
Age 2	-.11	.19	-.03	.24
Age 3	-.07	.28	.38	.35
Time waited	-.17	.12	.27†	.15
Role	-.05	.18	-.23	.23
Gender	-.02	.18	-.20	.23
Shift	-.10	.23	-.37	.29
Crowding	.01	.01	.01	.01
Cultural affiliation	-.22	.26		
Language accessibility (LA)	.07	.23		
Openness to diversity (OTD)	.66*	.26		
Culture X LA	.41	.37		
Culture X OTD	-.42	.39		
OTD X LA	-.56	.36		
Culture X LA X OTD	1.39*	.58		
Satisfaction			-.19*	.09
R^2^	.14		.09	

N = 214

† p < .10

* p < .05, ** p < .01.

Notes. Age 1 = 17–29, Age 2 = 30–49, Age 3 = 50–69; Age 1 is the reference age criterion; Unstandardized coefficients with standard errors (SE).

**Fig 2:**
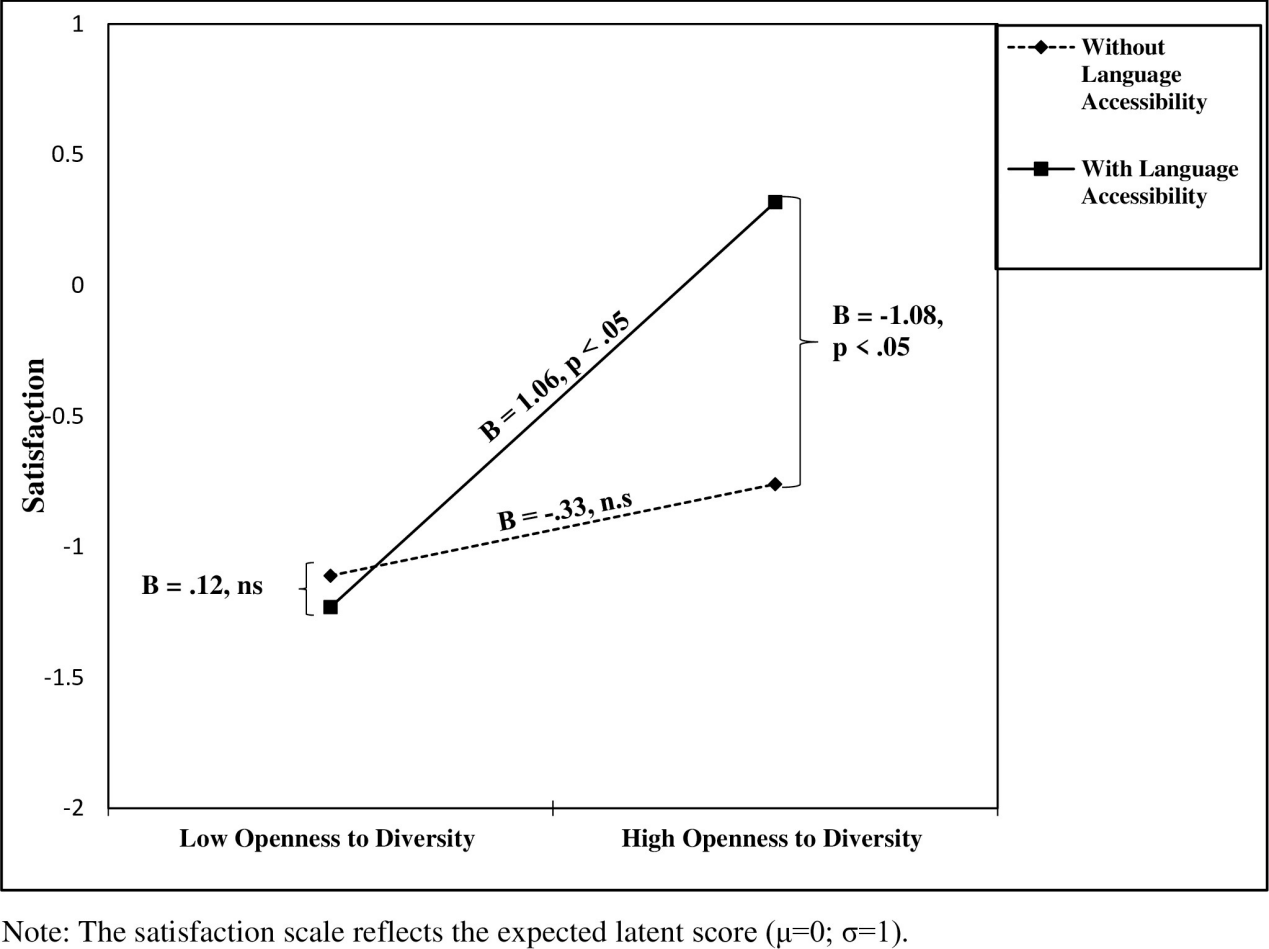
Interaction of care receivers’ openness to diversity and language accessibility on care receivers’ satisfaction for a cultural minority group (Israeli Arabs).

The results revealed a significant three-way interaction between care receivers’ OTD, language accessibility, and cultural affiliation, influencing satisfaction (B = 1.39, p < .05). To probe the nature of this interaction, we conducted a simple slopes analysis (following [88]), testing the interaction between OTD and language accessibility separately for the different cultural affiliation groups. The interaction was not significant for the Jewish participants (B = -.56, n.s.) and was significant for the Arab participants (B = 1.38, p < .05). Further analysis for the Arab participants revealed that care receivers’ OTD was positively related to satisfaction only among care receivers in the language accessibility group (B = 1.06, p < .05), but not among those who did not receive explanatory information in their native language (B = -.33, n.s.). Moreover, for Arab care receivers with high OTD (1 SD above the mean), those in the language accessibility group were more satisfied than those in the no-treatment group (B = -1.08, p < .05). This effect was not found for those with low OTD (1 SD below mean; B = .12, n.s.; see Fig 2). These results support Hypothesis 1. Additionally, a negative relationship was found between care receivers’ satisfaction and their aggressive tendencies (B = -.19, p < .05), supporting Hypothesis 2.

Finally, to test Hypothesis 3, we analyzed the conditional indirect effect of care receivers’ OTD on their aggressive tendencies through satisfaction in four conditions—2: cultural group affiliation (Jewish/Arab) X 2: language accessibility (yes/no). The results showed an indirect effect only for Arabs in the language accessibility treatment group (B = -.21; 95% CI (-.58, -.03)). That is, for Arab care receivers, OTD was negatively and indirectly related to aggressive tendencies through satisfaction when they received explanatory information in their native language, but not when they did not (B = .06; 95% CI (-.10, .40); IMM = -.27; 95% CI (-.86, -.01)). Among Jews, no such effect was found, either in the treatment group, which was given explanatory information in Hebrew (B = -.02; 95% CI (-.21, .08)), or the control group, which did not receive such information (B = -.12; 95% CI (-.40, .01); IMM = .11; 95% CI (-.01, .45)). These results are consistent with Hypothesis 3.

## Discussion

Enhancing care receivers’ satisfaction and reducing aggressive tendencies has great economic and psychological benefits for care receivers, staff, and health care organizations.

Our study examines how the interaction between three cultural factors (care receivers’ OTD, cultural affiliation, and language accessibility) influences care receiver satisfaction and, in turn, aggressive tendencies. The results indicate that high OTD enhanced satisfaction and reduced aggressive tendencies only among care receivers who belonged to the cultural minority (Arabs), and only when information about ED processes was made accessible to them in their own language. Thus, a combination of language accessibility for ethnic minority care receivers, and high care receivers’ openness to cultural diversity, play a strong role in enhancing their satisfaction and reducing their aggressive tendencies.

Our findings offer several insights. First, most previous studies that explored the relationship between culture and satisfaction in health care systems examined only a single cultural factor (e.g., the relationship between cultural affiliation and satisfaction or between cultural competence and satisfaction [89, 90]. Our findings show that different aspects of culture interact with each other to explain care receivers satisfaction in the ED context. Hence, these results support the CuPS approach [31], and specifically the notion that individuals’ perceptions and behaviors in multicultural contexts are complex phenomena which derive from an interaction between cultural affiliation, individual competencies, and situational factors.

Second, we found that language accessibility (in the form of written information in their native language) heightens satisfaction among care receivers from a minority cultural group. Considering the various existing information delivery methods, written information is a low-cost, easy, and efficient way to inform care receivers about the administrative and medical procedures in the ED [91]; and we find this makes a meaningful contribution for cultural minority groups even though it is quite lean in social cues. Language accessibility that is richer in social cues (e.g., voice or video) is expected to strengthen the positive impact on ED outcomes even more [92]. Our findings thus suggest that ensuring accessibility to such information in the languages used by local ethnic minorities is an easy way for hospitals to improve their service to those populations. Interestingly, we found no effect of the information provided in Hebrew (the language of the majority in Israel) among members of the majority community, even though it is reasonable to assume that information delivery should improve satisfaction for all care receivers. Previous studies indicated that care receivers who do not master the ED’s majority language as their native language receive fewer explanations from the staff than those who do [42, 49]. This can be explained by the latter’s ability to access medical and administrative information directly, using their verbal capabilities in the majority language. Our study may imply that the effect of written information in a native language is more meaningful for the minority care receivers who are not sharing the same level of verbal capabilities in the majority language. Another possible explanation can be related to psychological safety. Receiving written information in a native language (that is not the majority language) can enhance a sense of familiarity and security, which leads to satisfaction [48, 50]. Such an effect can be less significant for individuals who use the majority language as their native language.

Our study also departs from much existing literature on culture and aggression in health care contexts in that we consider characteristics of care receivers that can facilitate satisfaction in a culturally diverse context. We found that the cultural competency of care receivers, in the form of OTD, plays a significant role in this process.

### Practical implications

From a practical perspective, our study support previous findings showing that low satisfaction levels among care receivers are an antecedent of aggressive tendencies in hospitals [93]. Our results suggest that ED managers can reduce such aggressive tendencies by investing resources in programs and procedures aimed at improving an atmosphere of cultural sensitivity (e.g., through language accessibility) in the ED. Our study demonstrates that delivering medical and administrative information to cultural minority groups via written material in different languages is an efficient, low-cost way to enhance care receivers’ satisfaction and reduce aggressive tendencies. Indeed, we found an effect even with just a single page of information distributed at the reception desk. Moreover, today’s technology allows for multiple ways of providing such information, from old-fashioned hard copies to instant messages and video clips sent directly to care receivers’ mobile phones. As noted earlier, means of communication that are richer in social cues, such as voice or video, are expected to strengthen the positive impact of language accessibility on ED outcomes.

### Limitations and future directions

Our research has several limitations that also present opportunities for future research. First, we examined care receivers’ aggressive tendencies rather than actual behavioral aggression. Although it is extremely difficult to collect data from aggressive care receivers, we call on scholars to meet this challenge in future research. Second, aspects of our setting limit the generalizability of our findings. For instance, we focus our spotlight on the ED. As we know, cultural diversity exists in other health care systems and in various workplaces and industries, and it would be interesting to explore these effects in such contexts. Similarly, our study was conducted in Israel, where most minority ethnic groups—the source of cultural diversity examined in this study—are natives (e.g., Israeli Arabs) and not immigrants. Thus, our findings could be less relevant to subgroups of immigrants, who are known to be high in openness to diversity, as immigration by itself implies openness to different cultures. We therefore recommend that future research explore similar models in immigrant groups. In addition, we call for future studies to explore the role of culture in a broader sense, including the interplay between different aspects of national (or ethnic) culture, organizational culture, and professional or occupational culture with regard to care receivers’ perceptions and aggressive tendencies.

Last, we offer insights to the contribution of care receivers’ OTD to higher satisfaction and lower aggressive tendencies. A question that needs further exploration is how ED staff’ cultural characteristics interact with those of the care receivers. The ability of medical staff to interact effectively with diverse others is related to better communication with—and higher satisfaction among—care receivers [90]. Hence, when both care receivers and medical staff are motivated to interact with culturally diverse others, there should be an even greater increase in care receivers’ satisfaction and a concomitant decline in their aggressive tendencies. Another possibility is a compensation effect, when high OTD of medical staff may compensate for low levels of this characteristic among care receivers and vice versa. We recommend exploring these relationships especially for ED staff groups that have high interactions with care receivers (e.g., medical residents, nurses).

## Conclusion

In recent years, ED contexts are becoming more multicultural [32, 33]. Our results show that factors related to cultural affiliation, individual cultural characteristics, and the cultural situational setting can affect care receivers’ satisfaction and aggressive tendencies. Hence, health care systems that embrace a broad cultural perspective may reap benefits in the form of higher satisfaction among care receivers, and lower aggression against medical staff.

## Supporting information

S1 File Survey items -English. (DOCX)

S2 File Helsinki English. (DOCX)

S3 File Questionnaires in original languages. (DOCX)

S4 File Appendix A_ defining care receivers. (DOCX)
