# Supplementary material for: The influence of culture on care receivers’ satisfaction and aggressive tendencies in the emergency department
Source: PLoS One. 2021 Sep 2;16(9):e0256513. doi: 10.1371/journal.pone.0256513 (PMC8412260; doi:10.1371/journal.pone.0256513)
Supplement: S1 File — (DOCX) [file pone.0256513.s001.docx]

**List of Measures**

**Date____ Time______ #___________**

Openness to diversity (OTD) ( based on Hobman, Bordia & Gallois’s , 2003)

**To what extent do the following statements describe you accurately?**

I often spend time with people from cultural groups other than my own

I enjoy working with people from different ethnicity or culture

I make an extra effort to listen to people from different cultures

I enjoy working with people with different cultural values and motives

I enjoy learning from people with different cultural values and motives

Satisfaction (based on Glynn, Mangold, and Babaku's ,1991)

**Please state your impression of the emergency room staff (1-7)**

I perceive medical staff as willing to help me

I perceive medical staff as knowledgeable in their field

I perceive medical staff as polite

I felt confident in my communication with the staff

Aggression Tendencies ( Efrat-Treister et al., 2020).

**What do you think the chances of someone in the room behaving like this? (Obviously you can't know, please estimate the odds only)**

Curse a medical caregiver

Hit a medical caregiver

Harm hospital property

Bang on a table

Insult a medical caregiver

Yell at a medical caregiver

Sue the hospital

Insult the family of the medical caregiver in public

Role (patient or escort)

Gender_______

Age______
