# Supplementary material for: The influence of culture on care receivers’ satisfaction and aggressive tendencies in the emergency department
Source: PLoS One. 2021 Sep 2;16(9):e0256513. doi: 10.1371/journal.pone.0256513 (PMC8412260; doi:10.1371/journal.pone.0256513)
Supplement: S2 File — (DOCX) [file pone.0256513.s002.docx]

**Date: October 10th, 2016 To:**

**Dr. Vladimir Zeldtz**

**Emergency Medicine Department**

**Soroka Medical Center**

Dear Researcher,

Your request for runing a clinical experiment was considered in the Helsinki commeetee meeting held on October 6th, 2016. Below is the revelant section of the meeting protocol:

1. **Experiment details:**
2. **פרטי הניסוי:**

| **Medical protocol number** | **Helsinki request number**  0126-16-SOR |
| --- | --- |
| **Experiment topic**  Cultural sensitivity and accessibility in health care organizations | |
| **Name of local representative** | **Name of research initiator**  Dr. Alon Lisak  - |

1. **Research files**

| File description | Committee decision | Date |
| --- | --- | --- |
| Research protocol - 1  Addition to approval _ 1 Research questionnaire for staff  Addition to approval _ 1 Research questionnaire for patients | Approved  Approved  Approved | 05/04/2016  21/09/2016  21/09/2016 |

**Meeting summary**

**Conditions and further requests:**

**The request has been approved**

**(Special) Research initiator must report results to health ministry**

**Committee decision: Approved**

**This is a special medical experiment, that can be approved by the medical center’s manager, without further approval of the health ministry.**
