# Supplementary material for: The influence of culture on care receivers’ satisfaction and aggressive tendencies in the emergency department
Source: PLoS One. 2021 Sep 2;16(9):e0256513. doi: 10.1371/journal.pone.0256513 (PMC8412260; doi:10.1371/journal.pone.0256513)
Supplement: S3 File — (DOCX) [file pone.0256513.s003.docx]

**S3 File. Questionnaires in original languages**

**תאריך:_______ שעה:________ מספר מדבקה:_______**

**שאלון למקבלי שירות רפואי במיון**

שלום רב, המחקר הנוכחי בוחן מאפיינים התנהגותיים ואישיותיים המשפיעים על התנהגויות מטופלים ומלווים כלפי צוות המלר"ד. המחקר נערך על ידי ד"ר אלון ליסק וד"ר דורית אפרת-טרייסטר מהמחלקה לניהול באוניברסיטת בן גוריון בשיתוף ולאחר קבלת אישור ועדת האתיקה של המרכזים הרפואיים סורוקה וכרמל. הנך מתבקש/ת למלא שאלון קצר שאורך המילוי שלו הוא כ-10 דקות. המענה לשאלון התנדבותי והנך רשאי/ת שלא לענות על כל השאלות שבשאלון או על חלק מהן. איננו צופים שהשאלות יצרו אי-נוחות, אך בכל עניין ודבר הנך מוזמן לפנות לעוזרי המחקר בסמוך אליך, או לחוקרים אשר פרטי הקשר שלהם נמצאים בסוף השאלון. השאלון הינו **אנונימי**. הנך מתבקש להעביר לנו רק את מספר הנבדק מהמדבקה שקיבלת בכניסה למלר"ד, שלא כולל כל סימני זיהוי אישיים, על מנת לעקוב אחר האינטראקציות שלך עם גורמי המלר"ד השונים. כל המידע הקשור למחקר ישמר באופן מוצפן במחשבי החוקרים באוניברסיטה ורק להם תהיה גישה אליו. במידע לא יערך כל שימוש שאינו קשור למחקר עצמו. לשאלון זה אין תשובות נכונות או לא נכונות, ולפיכך חשובה לנו רק דעתך הכנה. ההוראות בשאלון מנוסחות בלשון זכר ומכוונות לשני המינים.

**בעצם מילוי השאלון הנך מצהיר/ה שהבנת את הכתוב מעלה ואת/ה מסכימ/ה להשתתף במחקר.**

**כמה זמן עבר מאז שהגעת לחדר המיון ועד עכשיו?** __שעות ו __דקות.

הקף בעיגול: האם אתה מטופל / מלווה מטופל ? אם אתה מלווה השלם: המטופל/ת הוא/היא ________ שלי.

כמה פעמים ביקרת בחדר המיון הזה זה בעבר?____________

האם **בביקור הנוכחי במיון** אתה/המטופל שאתה מלווה: נבדקת/טופלת על ידי אח/ות? הקף: לא / כן, ___פעמים.

נבדקת או טופלת על ידי רופא/ה? הקף: לא/ כן, ___ פעמים.

**במענה על כל השאלות הבאות השתמש בסקאלה הבאה:**

| **1**  **במידה מועטה מאד** | **2**  **במידה מועטה** | **3**  **במידה די מועטה** | **4**  **במידה בינונית** | **5**  **במידה די רבה** | **6**  **במידה רבה** | **7**  **במידה רבה מאד** |
| --- | --- | --- | --- | --- | --- | --- |

**האם מישהו תרגם עבורך את דברי אנשי הקבלה? ______ אם כן:**

| עד כמה הבנת את מה שתרגמו עבורך? | 1 | 2 | 3 | 4 | 5 | 6 | 7 |
| --- | --- | --- | --- | --- | --- | --- | --- |
| עד כמה המידע בשפתך היה ברור? | 1 | 2 | 3 | 4 | 5 | 6 | 7 |

**בחר את התשובה המתארת אותך בצורה הטובה ביותר כפי שהנך באמת:**

| לעיתים קרובות אני מבלה זמן עם אנשים מקבוצות תרבותיות אחרות משלי. | 1 | 2 | 3 | 4 | 5 | 6 | 7 |
| --- | --- | --- | --- | --- | --- | --- | --- |
| אני נהנה לעבוד עם אנשים מרקע תרבותי או אתני שונה משלי. | 1 | 2 | 3 | 4 | 5 | 6 | 7 |
| אני עושה מאמץ גדול להקשיב לאנשים הבאים מרקע תרבותי שונה משלי. | 1 | 2 | 3 | 4 | 5 | 6 | 7 |
| אני נהנה לעבוד עם אנשים שיש להם ערכים ומניעים שונים משלי. | 1 | 2 | 3 | 4 | 5 | 6 | 7 |
| אני אוהב ללמוד מאנשים שיש להם ערכים ומניעים שונים משלי. | 1 | 2 | 3 | 4 | 5 | 6 | 7 |

**אנא ציין את התרשמותך מעבודת הצוות שטיפל בך בחדר המיון:**

| אנשי צוות של בית החולים היו מוכנים לסייע לי. | 1 | 2 | 3 | 4 | 5 | 6 | 7 |
| --- | --- | --- | --- | --- | --- | --- | --- |
| אנשי הצוות של בית החולים היו בקיאים בתחומם. | 1 | 2 | 3 | 4 | 5 | 6 | 7 |
| אנשי הצוות של בית החולים היו מנומסים. | 1 | 2 | 3 | 4 | 5 | 6 | 7 |
| הרגשתי בטוח בתקשורת שלי עם אנשי הצוות. | 1 | 2 | 3 | 4 | 5 | 6 | 7 |

**מה לדעתך הסיכוי שמישהו בחדר יתנהג בצורה הבאה? (ברור שאינך יכול לדעת, אנא תעריך את הסיכויים בלבד)**

| יקלל. | 1 | 2 | 3 | 4 | 5 | 6 | 7 |
| --- | --- | --- | --- | --- | --- | --- | --- |
| יכה איש צוות רפואי. | 1 | 2 | 3 | 4 | 5 | 6 | 7 |
| יפגע ברכוש בית החולים. | 1 | 2 | 3 | 4 | 5 | 6 | 7 |
| ידפוק על השולחן. | 1 | 2 | 3 | 4 | 5 | 6 | 7 |
| יעליב איש צוות רפואי. | 1 | 2 | 3 | 4 | 5 | 6 | 7 |
| יצעק על איש צוות רפואי. | 1 | 2 | 3 | 4 | 5 | 6 | 7 |
| יגיש תביעה נגד בית החולים. | 1 | 2 | 3 | 4 | 5 | 6 | 7 |
| יעליב את משפחתו של איש צוות רפואי בפניו ולעיני כל. | 1 | 2 | 3 | 4 | 5 | 6 | 7 |

**הפרטים הבאים הם למטרות מחקר בלבד ויישארו חסויים:**

שנת לידה_____ מגדר (הקף): זכר/נקבה מהי שפת האם שלך? ____________

אילו שפות אתה מדבר, ומה רמת השליטה שלך בשפות אלו?

שפה_______ רמת שליטה________ שפה_______ רמת שליטה________

שפה_______ רמת שליטה________ שפה_______ רמת שליטה________

כמה שנות לימוד יש לך?_____(כולל לתואר גבוה) מה המצב הסוציואקונומי שלך? מעל הממוצע /ממוצע/ מתחת לממוצע

מהי הדת שלך?________ מהי רמת הדתיות שלך? (הקף) :אתאיסט/חילוני/מסורתי/דתי/דתי מאד.

**التاريخ:_______ الساعة:________ رقم اللاصقة:_______**

**استمارة لتلقي خدمة طبية في غرفة الطوارئ**

مرحبًا،

يفحص البحث الحالي خصائص سلوكية وشخصية تؤثر في تعامل متلقي العلاج ومرافقيهم مع الطاقم في غرفة الطوارئ. يجري دكتور ألون ليسك ودكتور دوريت إفرات - تريستر من قسم الإدارة في جامعة بن غوريون البحث معا وبعد مصادقة لجنتَي الأخلاقيات في المركزَين الطبيَين سوروكا والكرمل. نطلب منكَ/ منكِ تعبئة الاستمارة القصيرة التي تستغرق نحو 10 دقائق. الإجابة عن الأسئلة طوعية ويُسمح لك/لكِ عدم الإجابة عن كل الأسئلة المدرجة في الاستمارة أو على جزء منها. لا نتوقع أن تثير الأسئلة انزعاجا، ولكن يمكنك التوجه حول أي سؤال إلى مساعدي البحث الموجودين قريبا منك أو إلى الباحثين الذين تظهر تفاصيل الاتصال بهم في نهاية الاستمارة. الاستمارة مجهولة الهوية. نطلب منك أن تسجل رقم المفحوص الذي حصلت عليه عند دخولك إلى غرفة الطوارئ، الذي لا يتضمن أية علامات تعريف شخصية، بهدف متابعة التفاعل بينك وبين الجهات المختلفة في غرفة الطوارئ. سيُحتفظ بكافة المعلومات ذات الصلة بالبحث بشكل مشفّر في أجهزة حاسوب الباحثين في الجامعة وسيكون في وسع الباحثين الوصول إليها وحدهم. لن تسُتخدم المعلومات لأهداف أخرى سوى أهداف البحث. لا توجد "إجابات صحيحة" أو "إجابات خاطئة" في هذه الاستمارة، لذلك يهمنا رأيك الصريح فحسب. التعليمات في الاستمارة مكتوبة بلغة المذكر ولكنها تهدف إلى كلا الجنسين على حد سواء.

**مع تعبئتكَ/تعبئتكِ هذه الاستمارة أنتَ/أنتِ تصرح/تصرحين أنكَ/أنكِ فهمتَ/فهمتِ المكتوب أعلاه وتوافق/وتوافقين على المشاركة في البحث.**

**كم من الوقت مر منذ أن وصلت إلى غرفة الطوارئ وحتى الآن؟** __ساعة/ ساعات و __دقيقة/ دقائق.

**كم من الوقت مر منذ أن دخلت إلى غرفة الطوارئ وحتى تحدثت مع مهني يتحدث بلغتك الأم؟** __ساعة/ ساعات و __دقيقة/ دقائق.

أحط بدائرة: هل أنت متلقي العلاج / المُرافِق لمتلقي العلاج؟‎ ‎إذا كنت المُرافِق فأكمل: متلقّي / متلقية العلاج هو/ هي ________.

كم مرة زرت غرفة الطوارئ في الماضي؟____________

هل في **الزيارة الحالية في غرفة الطوارئ**: فحصك/ عالجك ممرض/ ممرضة؟ أحط: نعم/ لا، ___ مرة/ مرات.

فحصك أو عالجك طبيب‏/ة؟ أحط: نعم/ لا، ___ مرة/ مرات.

**عند الإجابة عن الأسئلة التالية استخدم السلم التالي:**

| **‎1‎**  **بدرجة قليلة جدا جدا** | **‎2‎**  **بدرجة قليلة** | **‎3‎**  **بدرجة قليلة جدا** | **‎4‎**  **بدرجة متوسطة** | **‎5‎**  **بدرجة كبيرة جدا** | **6**  **بدرجة كبيرة** | **7**  **بدرجة كبيرة إلى حد بعيد** |
| --- | --- | --- | --- | --- | --- | --- |

**هل قام احد بترجمة كلام موظفي الاستقبال لك؟ _____ اذا كان الجواب نعم:**

| 1. الى أي درجة فهمت الترجمة التي قيلت لك؟ | 1 | 2 | 3 | 4 | 5 | 6 | 7 |
| --- | --- | --- | --- | --- | --- | --- | --- |
| ب. إلى أي درجة كانت المعلومات واضحة في لغتك؟ | 1 | 2 | 3 | 4 | 5 | 6 | 7 |

**اختر الإجابة التي تصفك بأفضل شكل في الحقيقة:**

| في أحيانِ كثيرة، أقضي وقتا طويلا مع أشخاص من حضارات أخرى تختلف عن حضارتي. | 1 | 2 | 3 | 4 | 5 | 6 | 7 |
| --- | --- | --- | --- | --- | --- | --- | --- |
| أتمتع بالعمل مع أشخاص ذوي خلفيّة حضارية تختلف عن حضارتي. | 1 | 2 | 3 | 4 | 5 | 6 | 7 |
| أبذل جهدا كبيرا للإصغاء إلى أشخاص ذوي حضارات أخرى تختلف عن حضارتي. | 1 | 2 | 3 | 4 | 5 | 6 | 7 |
| أتمتع بالعمل مع أشخاص ذوي قيم ودوافع أخرى. | 1 | 2 | 3 | 4 | 5 | 6 | 7 |
| أحب التعلم من أشخاص ذوي قيم ودوافع أخرى. | 1 | 2 | 3 | 4 | 5 | 6 | 7 |

**ترجى الإشارة إلى انطباعك من عمل الطاقم الذي عالجك في غرفة الطوارئ:**

| كان أفراد طاقم المستشفى مستعدين لمساعدتي. | 1 | 2 | 3 | 4 | 5 | 6 | 7 |
| --- | --- | --- | --- | --- | --- | --- | --- |
| كان أفراد طاقم المستشفى خبيرين في مجال عملهم. | 1 | 2 | 3 | 4 | 5 | 6 | 7 |
| كان أفراد طاقم المستشفى لطفاء معي. | 1 | 2 | 3 | 4 | 5 | 6 | 7 |
| شعرتُ بأمان أثناء التواصل مع أفراد الطاقم. | 1 | 2 | 3 | 4 | 5 | 6 | 7 |

**ما هو الاحتمال وفق رأيك أن يتصرف شخص في الغرفة بالشكل التالي؟** **(من الواضح أنك لست قادرا على المعرفة، يرجى أن تقدّر الاحتمالات فقط)**

| يشتم (يسبّ). | 1 | 2 | 3 | 4 | 5 | 6 | 7 |
| --- | --- | --- | --- | --- | --- | --- | --- |
| يضرب أحد أفراد الطاقم. | 1 | 2 | 3 | 4 | 5 | 6 | 7 |
| يُلحق ضررا بممتلكات المستشفى. | 1 | 2 | 3 | 4 | 5 | 6 | 7 |
| يطرق على الطاولة. | 1 | 2 | 3 | 4 | 5 | 6 | 7 |
| يُهين أحد أفراد الطاقم. | 1 | 2 | 3 | 4 | 5 | 6 | 7 |
| يصرخ على أحد أفراد الطاقم. | 1 | 2 | 3 | 4 | 5 | 6 | 7 |
| يقدّم دعوى ضد المستشفى. | 1 | 2 | 3 | 4 | 5 | 6 | 7 |
| يُهين عائلة أحد أفراد الطاقم أمامه وأمام الجميع. | 1 | 2 | 3 | 4 | 5 | 6 | 7 |

**هذه التفاصيل معدّة لأهداف البحث فقط وستظل سرية:**

سنة الولادة _____ الجندر (أحط): ذكر/أنثى ما هي لغتك الأم؟ ____________

ما هي اللغات التي تتحدثها، وما هو مستوى تمكنك منها؟

اللغة _______ مستوى التمكن ________ اللغة _______ مستوى التمكن ________

اللغة _______ مستوى التمكن ________ اللغة _______ مستوى التمكن ________

كم سنة تعلمت؟_____(بما في ذلك تعليم للقب عال) ما هو وضعك الاجتماعي - الاقتصادي؟ أعلى من المعدل/ متوسط/ أقل من المتوسط.

ما هي ديانتك؟________ إلى أية درجة أنت متديّن؟ (أحط) :مُلحد/ علماني/ محافظ/ متديّن/ متديّن جدا.
