# Supplementary material for: The influence of culture on care receivers’ satisfaction and aggressive tendencies in the emergency department
Source: PLoS One. 2021 Sep 2;16(9):e0256513. doi: 10.1371/journal.pone.0256513 (PMC8412260; doi:10.1371/journal.pone.0256513)
Supplement: S4 File — (DOCX) [file pone.0256513.s004.docx]

**Appendix A: Defining Care Receivers**

In Israel, standard policy in any ED is that each patient may be escorted by one individual. Israel is a geographically small and collectivistic society [1]. Hence, most patients who need to attend an emergency department are accompanied by first-degree relatives (an adult child, sibling, spouse, or parent). This was the case in our study, where 88% of the escorts were close relatives of patients. Because close relatives tend to be highly invested in the patient's health, previous studies that explored aggression in Israeli EDs consider both patients and their escorts as one group of care receivers (e.g., [2, 3]). This rationale is also supported by findings from a range of countries that point to similar involvement of patients and their escorts (mostly relatives) in aggression against ED caregivers (e.g., Australia [4]; Italy [5], the USA [6], and China [7]), and by findings from other countries where escorts have been found to contribute meaningfully to such aggression (e.g., Ireland [8], South Korea [9] and South Africa [10]). These cumulative findings lead scholars to conclude that although the perpetrators of aggression against ED caregivers are varied, patients and their relatives can be identified as the largest perpetrator group (e.g., [5, 10]). In our study, we follow this accumulated knowledge and consider both patients and escorts as care receivers.

To assure that the care receiver role (patient or escort) did not change the relationship between our model variables, we controlled for this role during our analyses. For this purpose, we used structural equation modeling (SEM), similar to the main analyses in our study, but entering the care receiver role (patient/escort) as an additional factor in our model. We tested whether this factor interacts with openness to diversity, language accessibility and cultural affiliation to affect satisfaction, and with satisfaction to affect aggressive tendencies. All interactions were non-significant (p>.10). Hence we conclude that participants' role (whether patient or escort) does not change the relationships among the study variables. Additionally, independent sample t-test found no significant difference between the aggressive tendency scores of patients and escorts (p>.10). Last, the standard deviation of both groups was similar (1.65 and 1.59 for patients and escorts, respectively).

We also conducted a preliminary analysis to explore whether the nature of the relationship between escorts and patients (namely, escort type) led to different levels of aggressive tendencies. Specifically, we examined two questions: a) Do different relationships lead to different levels of aggressive tendencies? And b) do these types of relationships interact with patients’ medical conditions (as perceived by the escort) to affect the escorts’ aggressive tendencies?

To resolve these questions, we first categorized the nature of the relationship between escorts and patients into six different types (as a reminder, all patients and escorts were adults). In the study (129 escorts), the escorts were as follows: a son or daughter (55 patients); a spouse (29 patients); a sibling (19 patients); a parent (10 patients); another relative (4 patients); or others (12 patients).

To explore the first question, we conducted an ANOVA. The differences between the categories in escorts’ aggressive tendency scores were insignificant (F=.54, n.s.), indicating that different types of escorts do not differ in their aggressive tendencies.

To address the second question, we conducted an ANCOVA to test whether escort type interacts with the medical condition of the patient (as perceived by the escort). Escorts rated the patient’s medical condition on a 6-point Likert-type scale (1= not severe, 6= critical; M=2.91, SD= 1.36). The interaction effect between the type of escort and the perceived medical condition of the patient was insignificant (F=.47, n.s.). Levene's test for equality of error variances was also not significant.

In sum, we did not find significant differences related to aggressive tendencies between escorts and patients and within the group of escorts. These results justify defining both patients and escorts as care receivers in the present study.

**References**

1. House RJ, Hanges PJ, Javidan M, Dorfman PW, Gupta V, editors. Culture, leadership, and organizations: The GLOBE study of 62 societies. Sage; 2004.
2. Efrat-Treister D, Moriah H, Rafaeli A. The effect of waiting on aggressive tendencies toward emergency department staff: Providing information can help but may also backfire. PLoS One. 2020; 15(1): e0227729.
3. Cheshin A, Rafaeli A, Eisenman A.Encountering anger in the emergency department: Identification, evaluations and responses of staff members to anger displays. Emerg Med Int. 2012 Jan 1.ID 603215. doi:10.1155/2012/603215.
4. Lyneham J. Violence in New South Wales emergency departments. Aust J Adv Nurs. 2001;18(2):8-20.
5. Cannavò M, La Torre F, Sestili C, La Torre G, Fioravanti M. Work related violence as a predictor of stress and correlated disorders in emergency department healthcare professionals. Clin Ter. 2019;170(2):e110-23.
6. Gates D, Ross CS, McQueen L. Violence: Recognition, management and prevention. J Emerg Med. 2006;31(3):331-337.
7. Jiao M, Ning N, Li Y, Gao L, Cui Y, Sun H, et al.. Workplace violence against nurses in Chinese hospitals: A cross-sectional survey. BMJ Open. 2015;5(3):e006719
8. Ryan D, Maguire J. Aggression and violence – a problem in Irish accident and emergency departments? J Nurs Manag. 2006;14(2):106-115.
9. Jeong IY, Kim JS. The relationship between intention to leave the hospital and coping methods of emergency nurses after workplace violence. J Clin Nurs. 2018;27(7-8):1692-1701.
10. Kennedy M, Julie H. Nurses’ experiences and understanding of workplace violence in a trauma and emergency department in South Africa. Health SA. 2013;18(1): 1-9.
